# Polybenzimidazole Aerogels with High Thermal Stability and Mechanical Performance for Advanced Thermal Insulation Applications

**DOI:** 10.1021/acsami.5c05737

**Published:** 2025-05-26

**Authors:** Christos Pantazidis, Željko Tomović

**Affiliations:** Polymer Performance Materials Group, Department of Chemical Engineering and Chemistry, Interactive Polymer Materials Research Center (IPM) and Institute for Complex Molecular Systems (ICMS), Eindhoven University of Technology, Eindhoven MB 5600, The Netherlands

**Keywords:** polybenzimidazole, aerogels, insulation, thermal insulation, thermal stability

## Abstract

Advancements in the development of high-performance organic aerogels are essential for cutting-edge thermal insulation applications, where lightweight and durable structures, low thermal conductivity, and exceptional thermal stability are critical requirements. In this work, we present thermally stable and mechanically robust organic aerogels based on cross-linked benzimidazole-rich structures, specifically designed for thermal insulation. These aerogels exhibit a combination of valuable properties, including low density, large specific surface area and high porosity. Their tortuous mesoporous structures effectively reduce heat transfer by limiting gas-phase conduction, resulting in thermal conductivities as low as 23.9 mW m^–1^ K^–1^. This is coupled with a remarkable resistance to thermal decomposition (Td_1%_ > 500 °C), surpassing the stability of the original polymer precursor (OPBI). Additionally, the strong polymer network, reinforced by both covalent and noncovalent interactions, provides exceptional mechanical strength, allowing the aerogels to withstand substantial loads without fracturing. This unique combination of low density, high porosity, robust mechanical performance, and superior thermal stability makes these aerogels highly promising for demanding thermal insulation applications, such as thermal protection for space exploration vehicles, fire-resistant suits, and EV battery insulation.

## Graphical abstract

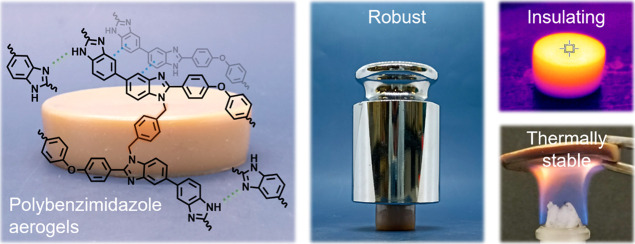

## 1 Introduction

Organic aerogels are an emerging class of porous solid materials characterized by low density, high open porosity, and a large specific surface area.^1^ Their tunable chemistry allows for functionalization and tailored molecular design, enabling diverse applications in catalysis, drug delivery, adsorption and energy storage.^2–5^ One of their most prominent applications is thermal insulation, where they outperform conventional materials such as polystyrene foams and glass wool,^6,7^ due to their ability to limit gas collisions within their nanostructured pores, effectively reducing the gas-phase thermal conductivity in the porous network through the Knudsen effect.^8,9^ However, despite the fact that organic aerogels offer chemical versatility and improved processability,^10^ they typically exhibit lower thermal stability and higher flammability compared to their inorganic counterparts.^1^ To address this limitation, thermally stable chemical moieties, such as imides,^11,12^ phosphazenes,^13,14^ cyanurates^15^ and isocyanurates,^16–19^ have been incorporated into their structures.

An alternative strategy for developing high-performance aerogels with enhanced thermal stability and mechanical strength is the formation of polymer networks through cross-linking inherently thermally stable polymers. Polybenzimidazole (PBI) and its’ copolymers are excellent candidates for fabricating such aerogels due to their high glass transition temperatures (*T*_g_) and outstanding thermal stability.^20,21^ Due to their thermal and mechanical resilience, PBI-based polymers are widely used in applications such as fuel cell membranes, firefighters’ protective gear, space suits, and emerging space exploration technologies.^22–25^ PBI aerogels with substantial thermal stability were recently reported for their potential usage in aerospace thermal protection systems.^26^ Since their first appearance, other attempts to develop benzimidazole containing insulating aerogels have focused on either incorporating benzimidazole units to enhance the structural stability of scaffolds with additional chemical moieties^27,28^ or cross-linking PBI-containing polymers through coordination bonds.^29,30^ While such aerogels demonstrate favorable characteristics of thermal and mechanical stability, they typically exhibit higher densities and elevated thermal conductivities (29–70 mW m^–1^ K^–1^), impeding on their practical application as lightweight insulation materials.

In this study, we use a commercially available polybenzimidazole copolymer (OPBI) containing a flexible diphenyl ether unit in its backbone, which enhances its solubility and processability. Through covalent cross-linking of this polymer, we developed lightweight, highly porous aerogels showing high structural stability, which is achieved via both primary (covalent) and secondary (hydrogen bonding and π–π stacking) interactions. These aerogels demonstrate excellent mechanical robustness while maintaining very low density (<100 mg cm^–3^), exceptional thermal stability (Td_5%_ > 580 °C), and flame resistance. Moreover, they surpass previously reported PBI-based aerogels and several commercial products in thermal insulation, achieving thermal conductivities as low as 23.9 mW m^–1^ K^–1^. This unique combination of properties makes them highly promising candidates for demanding thermal insulation applications, including space exploration, electric vehicle battery insulation, and fire resistant protecting equipment.^31^

## 2 Experimental Section

### 2.1 Materials

Poly-2,2′-*p*-oxidiphenylene-5,5′-dibenzimidazole, OPBI (Fumion AM) was purchased from Fumatech. α,α′-Dibromo-*p*-xylene (DBX, ≥98%), benzimidazole (≥99%) dimethyl sulfoxide (DMSO) and dimethyl sulfoxide-*d*_6_ (DMSO-*d*_6_), were purchased from Merck Life Science NV. *N*,*N*-dimethylacetamide (DMAc), dimethylformamide (DMF), ethanol and acetone were purchased from Biosolve B.V. *N*-Methyl-2-pyrrolidone (NMP) was purchased from Vivochem. All reagents were used without further purification. Liquid CO_2_ (grade 2.7), N_2_ (grade 5.0), and He (grade 4.6) were purchased from Linde gas B.V.

### 2.2 Methods

#### 2.2.1 Chemical Characterization

The chemical structures of the monomers used for the model reaction and the resulting model compound, BIM, were identified by nuclear magnetic resonance (NMR) spectroscopy, performed on a Bruker UltraShield spectrometer (400 MHz for ^1^H NMR and 100 MHz for ^13^C NMR) at 25 °C using DMSO-*d*_6_ as the solvent.

FT-IR analysis was conducted using a Thermo Fisher Scientific Nicolet iS20 spectrometer equipped with an Attenuated Total Reflection (ATR) accessory. Spectral data were collected over the range of 4000–450 cm^–1^.

#### 2.2.2 Physical and Structural Characterization

For the morphological characterization of the aerogels, cylindrical monoliths with the size of approximately 25 mm in diameter and 15 mm in height were used.

The bulk densities of the cylindrical monoliths were calculated as follows: ρ_b_ = m_OPBI‑A_/*V*_OPBI‑A_, where m_OPBI‑A_ is the mass of the aerogel and *V*_OPBI‑A_ = π*r* ^2^ *h* (*r* = radius and *h* = height) is the volume of the aerogel.

The Tristar II Plus instrument was used to evaluate the specific surface area and pore size distribution of the prepared aerogels, employing Brunauer–Emmett–Teller (BET) theory and the Barrett–Joyner–Halenda (BJH) model, respectively, through nitrogen physisorption at 77 K. Nitrogen grade 5.0 was selected for measuring physisorption isotherms. Prior to measurement, the samples were outgassed at 80 °C for 2 h.

The skeletal density and porosity of the samples were assessed using the AccuPyc II 1345 gas pycnometer, using Helium grade 4.6.

Scanning electron microscopy (SEM) images were obtained for morphological characterization of the aerogels using the FEI Quanta 200 3DFEG at an acceleration voltage of 10 kV. Prior to imaging, the samples underwent gold sputtering (40 mA, 40 s). ImageJ software was used to measure the diameter of worm-like fibers.

Compression tests were performed with a compression testing equipment (ZwickRoell Materials Testing Machine, Zwicki Z2.5/TN). The compressive modulus was calculated between 0.05% and 0.25% of deformation ratio.

#### 2.2.3 Thermal Characterization

For thermal conductivity, thermographic images and flame resistance tests, larger samples with a diameter of 55 mm and a height of 10 mm were prepared. The experimental procedures remained consistent with the smaller monoliths, with the only modification being the adjustment of the mold size.

OPBI-As thermal properties were assessed with thermogravimetric (TGA) analysis, which was conducted using a TGA 550 instrument (TA Instruments) under nitrogen, with a heating rate of 10 °C min^–1^, ranging from 40 to 800 °C. Samples of approximately 10 mg were used.

Thermal conductivity measurements were conducted using a heat flow meter (Thermtest Inc., HFM-25) at 20 °C and 45–50% humidity, in accordance with the ASTM C518 international standard. The instrument was calibrated prior to measurement, using EPS 1450E as a reference material (31.8 ± 0.1 mW m^–1^ K^–1^). The samples were placed between thermal plates maintained at stable temperatures of 10 and 30 °C, respectively, to allow the measurement of heat flux. To ensure proper contact with the thermal plates, the aerogel samples were carefully flattened with sandpaper, resulting in samples approximately 8 mm thick.

An infrared camera (Fluke Ti32) was used to record the thermographic images and the real-time temperatures of the aerogels. The samples were placed on a hot stage (120 °C) and cold stage (−20 °C) for 10 min with external environment at 20 °C and relative humidity of 45–50%.

### 2.3 Model Reaction

The model reaction was performed by adding 200 mg (0.76 mmol, 1 equiv) of α,α′-dibromo-*p*-xylene (DBX) to 895 mg (7.6 mmol, 10 equiv) of benzimidazole in 10 mL of *N*,*N*-dimethylacetamide (DMAc). The reaction mixture was stirred at 70 °C for 2 h, after which the resulting model compound, *p*-Bis­(benzoimidazolyl)-methylbenzene (BIM), was precipitated in 100 mL of cold water. The precipitate was recovered by filtration, thoroughly washed with water and dried overnight in a vacuum oven at 80 °C, yielding BIM as a white solid (200 mg, 6.5 mmol, 86% yield). ^1^H NMR (400 MHz, DMSO-*d*_6_): δ = 8.38 (s, 2H), 7.65 (dt, 2H), 7.45 (dt, 2H), 7.26 (s, 4H), 7.18 (dt, 4H), 5.44 (s, 4H) ppm. ^13^C NMR (100 MHz, DMSO-*d*_6_): δ = 144.6, 143.7, 136.9, 133.9, 128.2, 123.1, 122.3, 119.9, 111.2, 47.7 ppm (Figure S1).

### 2.4 OPBI Aerogel Preparation

The OPBI aerogels (OPBI-A) presented in this study were synthesized by reacting poly-2,2′-*p*-oxidiphenylene-5,5′-dibenzimidazole (OPBI) with α,α′-dibromo-*p*-xylene (DBX) as a cross-linker in DMAc. Various OPBI concentrations and DBX molar ratios relative to OPBI monomeric units were explored. Here, the preparation of OPBI-A1 is described, with all other samples following the same protocol, differing only in precursor quantities as detailed in Table S1. For the preparation of OPBI-A1, 400 mg of OPBI were added to 9 mL of DMAc and then placed in an oven at 100 °C for 5 h, with occasional mixing using a vortex mixer, ultimately yielding a dark brown, viscous solution. Subsequently, 26.4 mg of DBX were dissolved in 1 mL of DMAc at room temperature, added to the OPBI solution and vigorously mixed to achieve homogeneity. The mixture was left to react at 100 °C for 2 h, forming a stable organogel, after which the temperature was lowered to 70 °C and the gel was aged overnight (15 h) to ensure maximum conversion. The resulting organogel underwent solvent exchange, initially with 0.05 M NaOH (aq.) solution, followed by water washing, and was then transferred into an acetone bath (∼100 mL, approximately 10 times the volume of the original solution) where it was stirred overnight to reach equilibrium. The acetone was then replaced with fresh acetone and this process was repeated two times to ensure complete solvent exchange. Finally, the acetone-containing organogel was subjected to supercritical drying using CO_2_ at 100 bar and 60 °C, as described in the Supporting Information (Scheme S1).

## 3 Results and Discussion

### 3.1 Model Reaction

Prior to aerogel synthesis, a reaction was conducted between DBX and benzimidazole to form the BIM model compound (Figure 1). While this reaction is typically highly efficient in the presence of a basic catalysts,^32,33^ our objective was to determine whether it could proceed under mild conditions without a catalyst. This reaction served as a model for the cross-linking process between DBX and the benzimidazole units of OPBI (Figure 2), which was employed in this work to form the polymer network used for aerogel fabrication. As described in the experimental section, the precursors were mixed and allowed to react in DMAc at 70 °C. After 2 h, a small aliquot was extracted and analyzed by ^1^H NMR, which revealed a complete shift of the cross-linker’s benzylic protons (4.70 ppm), confirming the full consumption of DBX (Figure S1). The resulting model compound (BIM) was precipitated in water, isolated in high yield (86%) and exhibited a characteristic peak at 5.44 ppm corresponding to the formation of benzylic bridges between DBX and benzimidazole as confirmed by ^1^H NMR and ^13^C NMR (Figures 1 and S1).

**Figure 1:**
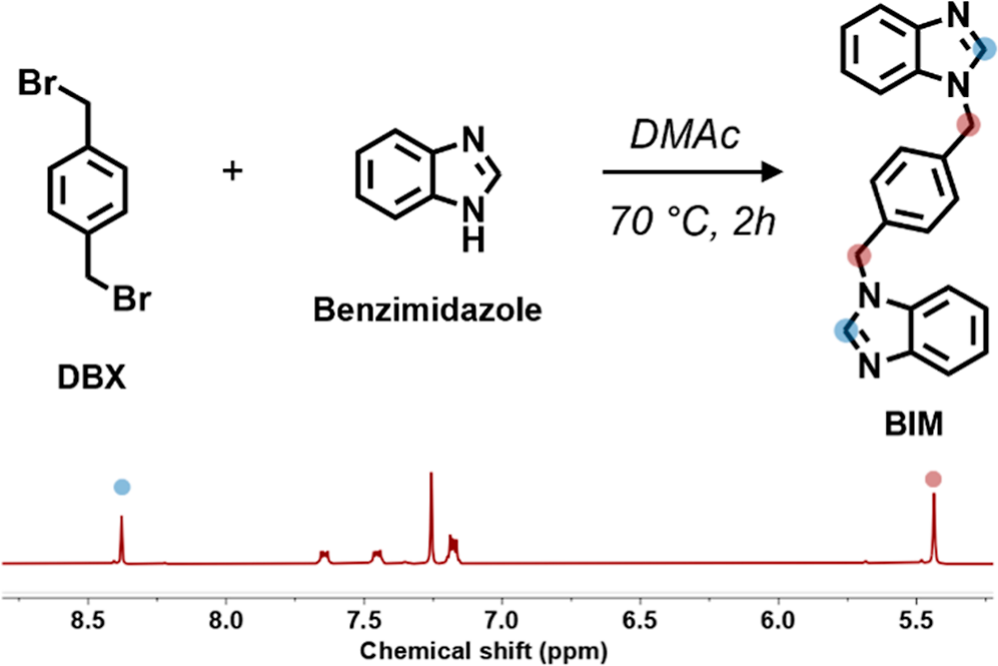
Model reaction between DBX and benzimidazole, with the corresponding ^1^H NMR of the model compound, BIM.

**Figure 2:**
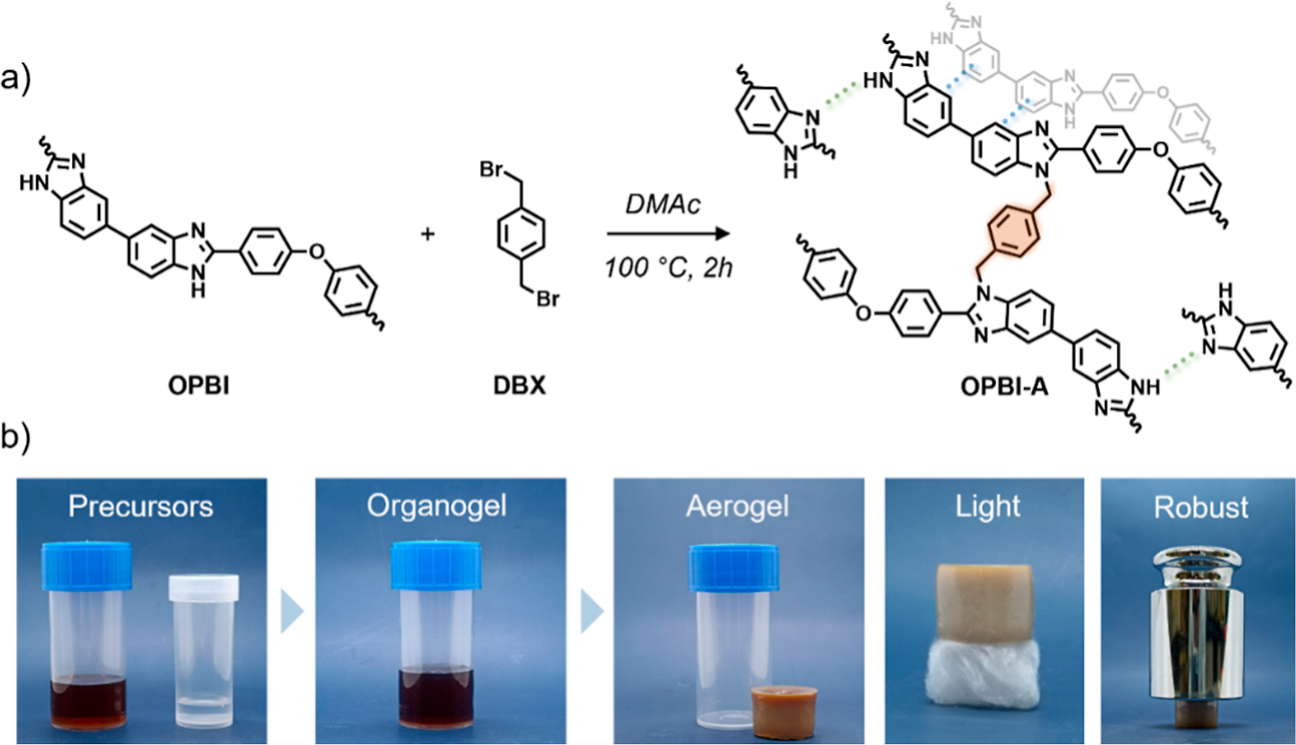
(a) Aerogel formation (OPBI-A) by the cross-linking reaction of poly-2,2′-*p*-oxidiphenylene-5,5′-dibenzimidazole (OPBI) with α,α′-dibromo-*p*-xylene (DBX). The network is stabilized through covalent and noncovalent interactions. (b) Representative photographs of the aerogel synthesis process. In the final two images, the light and robust nature of the aerogels is highlighted, as the same sample that rests on a piece of cotton can also withstand more than 2500 times its own weight (1 kg).

### 3.2 Design and Fabrication of OPBI Aerogels

The OPBI aerogels (OPBI-A) were synthesized via a cross-linking reaction between the benzimidazole groups of the OPBI copolymer and DBX, a covalent cross-linker. Additional interactions, such as hydrogen bonding and π–π stacking, further contributed to the structural integrity of the polymer scaffold (Figure 2). OPBI was selected for this study because, in addition to its benzimidazole groups, it incorporates diphenyl units within its repeating structure, providing partial flexibility and improving solubility in organic solvents.

The aerogel synthesis followed a sequence of precursor mixing, organogel formation, aging, solvent exchange, and drying using supercritical CO_2_ (see Experimental section). While the model reaction with small molecules demonstrated that efficient cross-linking could be achieved at 70 °C (Figure 1), a higher temperature (100 °C) was employed during the initial gelation stage to reduce viscosity and ensure better homogeneity during the mixing and early stages of the reaction. For the aging process, the temperature was lowered to 70 °C, and the organogels were left overnight to react, ensuring maximum conversion. The solvent exchange process began with an aqueous solution of 0.05 M NaOH to neutralize the HBr byproduct, followed by water to remove excess base and salts. Acetone was then used for the final solvent exchange step before supercritical CO_2_ drying which is detailed in the Supporting Information (Scheme S1). The resulting aerogels retained approximately 95–98% of the combined weight of the original precursors and showed the disappearance of the FT-IR peak corresponding to C–Br bonds (608 cm^–1^, C–Br stretch), indicating that DBX was fully consumed as a cross-linking agent. Additionally, a relative decrease in the FT-IR signal associated with N–H stretching (3250–3400 cm^–1^) was observed after cross-linking (Figure S2).

In order to find the optimal conditions required for the synthesis of aerogels with low density, we performed a screening process using different organic solvents (DMSO, NMP, DMAc, DMF), varying OPBI concentrations (2, 4, and 6% w/v) and different DBX molar ratios (5 and 10%) relative to OPBI repeating units (Table S1). Organogels prepared with 2% w/v OPBI were generally unstable, exhibiting significant deformation and shrinkage during solvent exchange, prompting a shift to 4% w/v OPBI and 5% DBX molar ratio for subsequent trials. In this case, organogels formed in NMP and DMAc were sufficiently stable to undergo drying, but the resulting aerogels exhibited significant diameter shrinkage (>20%). To improve structural robustness, the cross-linker ratio was increased to 10%, which resulted in more stable organogels and reduced final shrinkage (16–18%). Additionally, samples with 6% w/v OPBI were prepared in NMP and DMAc, demonstrating excellent stability and shrinkage below 15%. While the characteristics of aerogels prepared in both solvents were comparable, DMAc-based samples exhibited superior surface homogeneity, leading to their selection for further study (Table S1).

### 3.3 Physical Properties and Morphology of OPBI Aerogels

The synthesized OPBI-As were thoroughly characterized to evaluate their morphological, thermal, and mechanical properties. As an initial step, linear shrinkage and bulk density were measured. The linear shrinkage values were notably lower than those typically reported in the refs 34–36 and fell within a similar range (≈13–16%). The OPBI-As exhibited low bulk densities; 68 mg cm^–3^ for OPBI-A1 and nearly identical densities for OPBI-A2 and OPBI-A3, at 92 and 93 mg cm^–3^, respectively. These results enable comparisons of properties related to both, the bulk density of the samples which can be controlled by the original solution concentration, and the impact of increasing cross-linking density through modulation of the DBX content. A comprehensive summary of their physical characteristics and morphology is provided in Table 1.

**Table 1:** Physical Properties of OPBI-As

sample name	OPBI concentration^a^ (g mL^–1^)	DBX molar ratio^b^ (%)	linear shrinkage^a^(%)	bulk density, ρ_b_ (mg cm^–3^)	skeletal density, ρ_s_ (g cm^–3^)	Porosity^c^(%)	specific surface area^d^ (m^2^ g^–1^)	pore volume^e^(cm^3^ g^–1^)	average pore width^e^ (nm)
OPBI-A1	0.04	10	16	68	1.37	95.0	593	1.30	10.8
OPBI-A2	0.06	5	13	92	1.30	93.1	724	1.53	10.9
OPBI-A3	0.06	10	13	93	1.33	93.2	639	2.51	15.9

a OPBI concentration during gelation.

b Relative to OPBI repeating unit.

c Calculated as follows: (1 – ρ_b/_ ρ_s_) × 100%.

d Extrapolated from adsorption isotherms using BET method.

e Refers to mesopores and was extrapolated from desorption isotherms, using BJH method.

Microstructural and morphological analysis were conducted using nitrogen physisorption and scanning electron microscopy (Figure 3). Overall, the OPBI-As exhibited a complex network of interconnected domains, resulting in highly tortuous, mesoporous structures. The skeletal density of the OPBI-As (ρ_s_) was determined via helium pycnometry, and these values were used to calculate the samples’ porosity, which exceeded 93% in all cases (Table 1).

**Figure 3:**
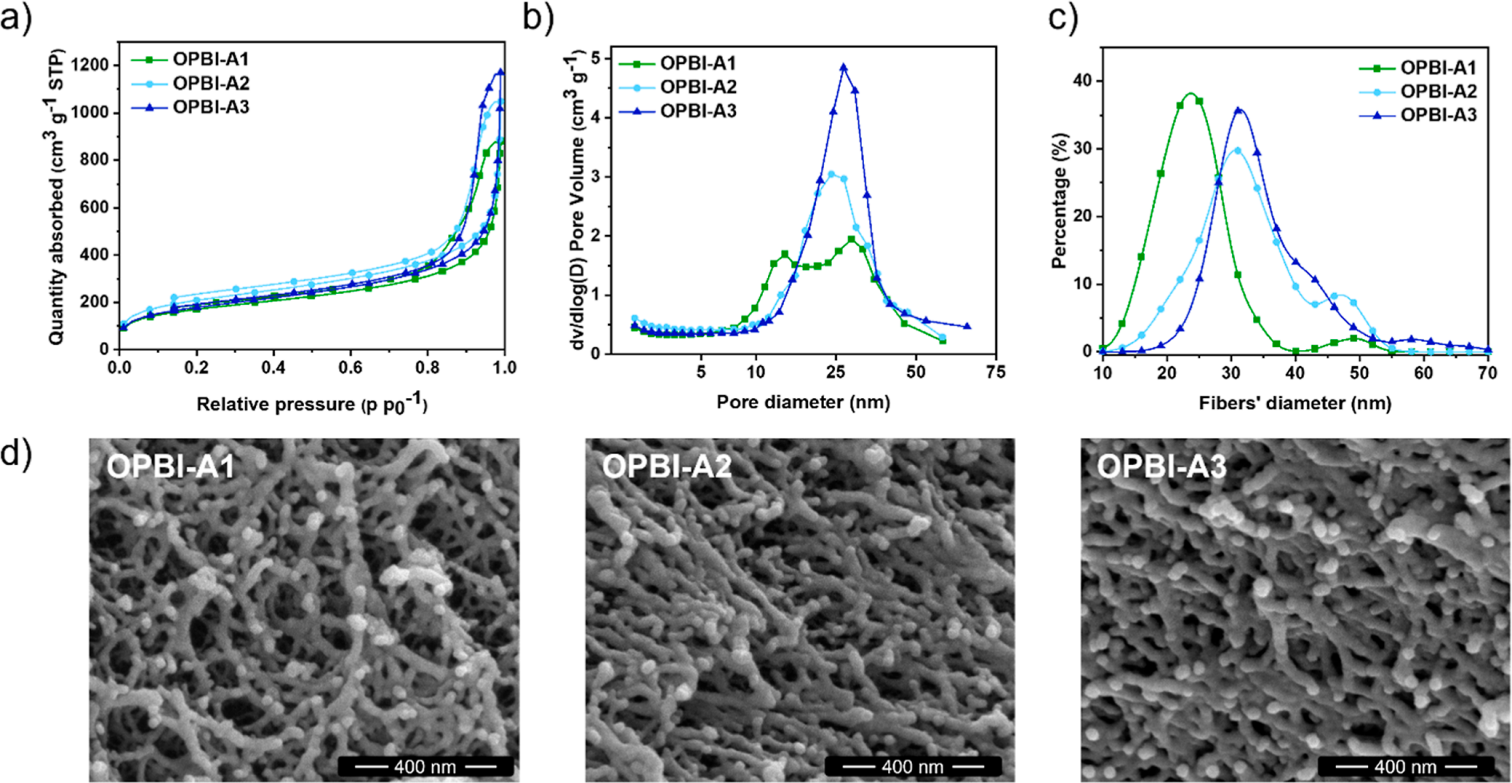
(a) N_2_ adsorption and desorption isotherms of OPBI-As at 77 K. (b) BJH pore size distribution of OPBI-As. (c) Fiber diameter distributions based on 100 fibers; SEM pictures and ImageJ software were used. (d) SEM pictures of OPBI-As (see Figure S3, Supporting Information, for range of magnifications).

The nitrogen adsorption–desorption isotherms (Figure 3a) displayed type IV curves, indicating the presence of mesoporous domains (2–50 nm).^37^ Specific surface areas were calculated using the Brunauer–Emmett–Teller (BET) method, with all samples exhibiting similar values in the range of approximately 600–700 m^2^ g^–1^. Notably, around 10% of the samples’ total surface area was attributed to microporous domains (<2 nm), which may arise from secondary interactions that create localized ordering or denser regions within the primary network, effectively creating smaller secondary pores. Additional evidence of hierarchical porosity is observed in the pore size distributions, where OPBI-A1 exhibits a distinct bimodal distribution with peaks at 14 and 30 nm (Figure 3b). This effect is less pronounced in OPBI-A2, which was synthesized with a higher initial OPBI concentration (6% w/v instead of 4% w/v) and displayed a broad distribution with two peaks at 24 and 33 nm. In OPBI-A3, where both a higher polymer concentration and increased DBX content were used, hierarchical porosity is almost entirely diminished.

SEM analysis further confirmed the intricate pore structures of the OPBI-As (Figure 3d). The SEM images revealed a worm-like morphology, with no visible heterogeneity or regions containing bulky particles or large voids (see Figure S3 for images at various magnifications). Additionally, an increase in the average fiber diameter was observed with higher OPBI concentrations during gelation. OPBI-A1 primarily exhibited a fiber diameter distribution with a mode value of approximately 24 ± 2 nm, while OPBI-A2 and OPBI-A3 showed broader distributions with higher mode values of 31 ± 3 nm (Figure 3c). The fact that OPBI concentration has a more pronounced effect on fiber diameter compared to the impact of the cross-linker ratio, indicates that secondary interactions, sensitive to polymer concentration, such as hydrogen bonding and π–π stacking, play a very important role in the formation of the OPBI network during gelation.

### 3.4 Thermal and Mechanical Properties of OPBI Aerogels

Thanks to their lightweight nature and high porosity, OPBI-A samples show great potential for thermal insulation applications. To evaluate their suitability as high-performance insulating materials, their thermal conductivity and mechanical properties were assessed, with the results discussed below and summarized in Tables 2 and 3.

**Table 2:** Thermal Conductivity and Mechanical Performance of OPBI-As

sample name	OPBI concentration^a^ (g mL^–1^)	DBX molar ratio^b^ (%)	bulk density, ρ_b_ (mg cm^–3^)	thermal conductivity (mW m^–1^ K^–1^)	compressive modulus^c^ (MPa)	compressive strength at 10% deformation^c^ (MPa)
OPBI-A1	0.04	10	68	23.9	0.89 ± 0.10	0.28 ± 0.01
OPBI-A2	0.06	5	92	26.7	1.19 ± 0.30	0.41 ± 0.05
OPBI-A3	0.06	10	93	29.7	1.27 ± 0.12	0.76 ± 0.12

a OPBI concentration during gelation.

b Relative to OPBI repeating unit.

c Average values using 3 samples.

**Table 3:** Thermogravimetric Data of OPBI Polymer and OPBI-A

sample name	decomposition onset Td_1%_ (°C)	Td_5%_ (°C)	char residue at 790 °C (wt %)
OPBI	471	596	54.5
OPBI-A1	509	582	82.6

The tortuous mesopore structures of the OPBI-As effectively limited heat conduction by enhancing the Knudsen effect, while their high porosity contributed to the overall lightweight nature of the material, resulting in low thermal conductivity values ranging from 23.9 to 29.7 mW m^–1^ K^–1^ (Table 2). Among them, OPBI-A1 exhibited the lowest thermal conductivity, attributed to its lower bulk density (68 mg cm^–3^) and higher porosity (95%). In contrast, samples with higher bulk densities, such as OPBI-A2 and OPBI-A3, exhibited higher thermal conductivity, which aligns with typical aerogel behavior, as increased density generally facilitates greater heat transfer through the solid framework.^38^ Furthermore, among samples with similar bulk density, OPBI-A3, which contained a higher DBX content—and consequently a greater degree of cross-linking—exhibited a higher thermal conductivity (Table 2). This may be attributed to enhanced thermal conduction through the denser skeletal framework of the more cross-linked polymer network,^39^ or to closer chain packing that promotes stronger nonbonding interactions (e.g., hydrogen bonding), which can also facilitate heat transfer through the solid matrix.^40^

Remarkably, the OPBI-A samples exhibited superior insulating properties compared to previously reported polybenzimidazole-based aerogels and were highly competitive with commercially available thermal insulation materials (glass wool, cork) and foams (EPS, XPS, PU) (Figure 4a). To further illustrate the thermal insulation capabilities of OPBI-A1, an infrared (IR) camera was used to monitor its response to temperature changes. A 10 mm-thick sample was placed on a hot and cold plate with temperatures of 120 and −20 °C respectively. After 10 min, the surface temperature at the midpoint of the aerogel was recorded. Owing to its low thermal conductivity, heat transfer through the sample remained minimal, resulting in only moderate surface temperature variations relative to the stage temperature. Moreover, the uniform temperature distribution confirmed the absence of thermal gaps or significant localized differences (Figure 4b).

**Figure 4:**
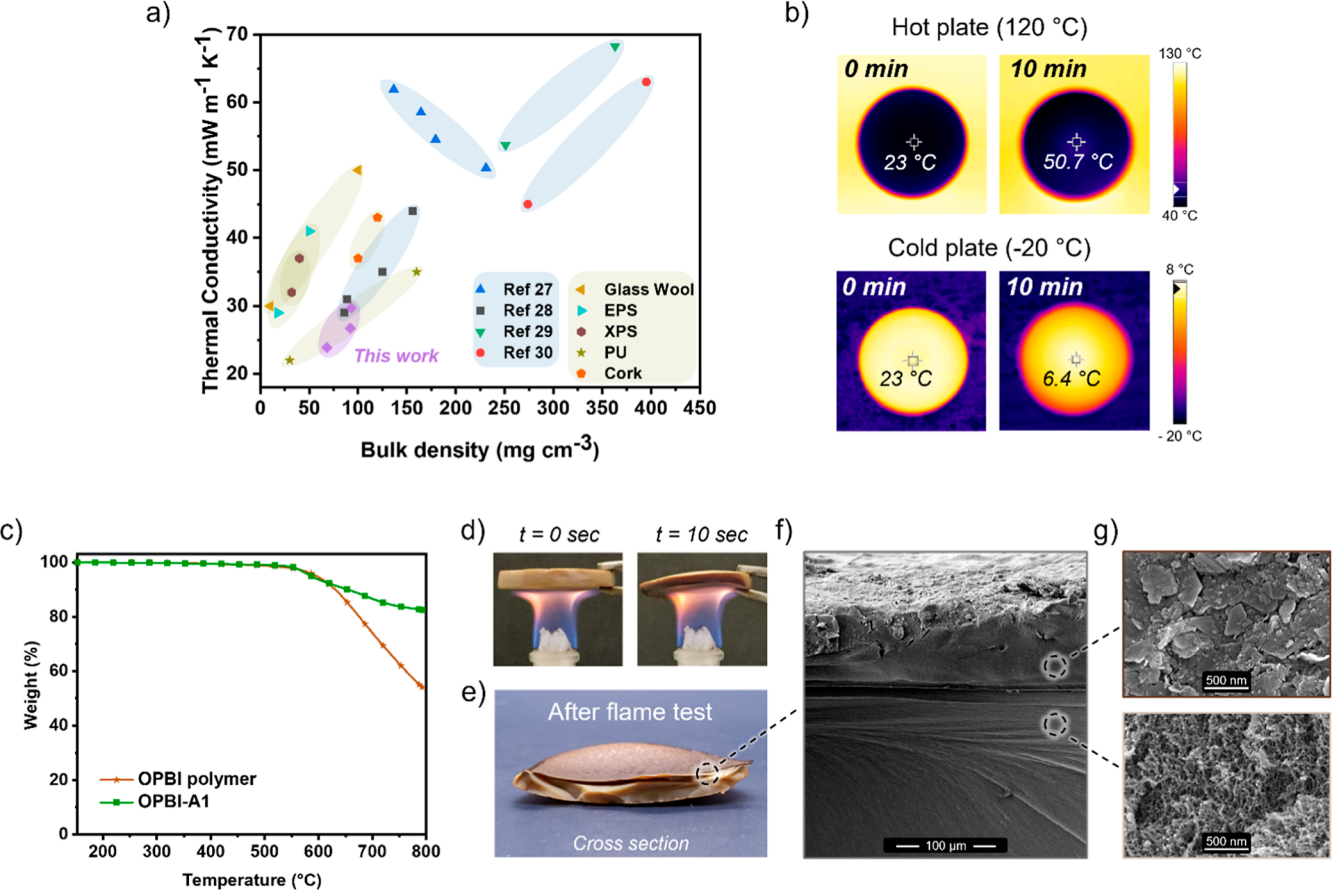
(a) Thermal conductivity values of OPBI-As in comparison of other benzimidazole based aerogels^27–30^ and commercial thermal insulation materials^6^ of ranging bulk densities. (b) Top-view IR images of OPBI-A1 placed on hot (120 °C) and cold (−20 °C) stage at the beginning and after 10 min (c) TGA curves under N_2_ flow. (d) OPBI-A1 subjected to ethanol flame. (e) Cross-section of OPBI-A1 after flame tests. (f) SEM picture of the OPBI-A1 cross-section after flame tests (100 μm bar). (g) SEM pictures of charred layer (top) and porous interior (bottom) of OPBI-A1 after flame tests.

Followingly, the thermal and flame stability of the best-insulating sample, OPBI-A1, was further examined to evaluate the potential of OPBI-As for insulation applications requiring high-temperature and flame resistance, such as building insulation, protective insulation for EV batteries, lightweight thermal protection suits and space exploration rovers. Thermogravimetric analysis (TGA) of OPBI-A1 revealed exceptional resistance to thermal decomposition, with the cross-linked aerogel structures exhibiting significantly greater stability than the original OPBI polymer. This was reflected in the higher onset decomposition temperature (Td_1%_) and greater char residue at 790 °C of OPBI-A1, as summarized in Table 3.

The high char residue observed during TGA measurements, prompted further evaluation of OPBI-A1’s flame resistance through a preliminary combustion test. A flat sample was exposed to an ethanol flame for at least 10 s and exhibited remarkable stability, showing no sustained combustion or smoke production (Figure 4d, Movie in Supporting Information). Notably, after the test, a cross-sectional cut revealed that the interior remained virtually unaffected, protected by a thin char layer formed during flame exposure (Figure 4e). SEM imaging further highlighted this self-protecting behavior, showing an approximately 100 μm thick compact charred layer on the aerogel’s surface, while the porous interior remained intact (Figure 4f,g). The observed flame resistance may be attributed to the formation of a stable pyrolyzed (char) layer, which acts as a physical barrier. This layer limits heat and mass transfer, thereby suppressing thermal feedback and fuel diffusion into the interior porous structure, preserving its morphology even after flame exposure.^31^ This self-generated protective barrier underscores the exceptional potential of these aerogels as high-performance insulating materials, particularly for applications requiring enhanced thermal stability.

Beyond their low thermal conductivity and thermal stability, robust mechanical performance is essential for organic aerogels to ensure practical usability, including handling, resistance to surface pressure, and durability under mechanical stress. Remarkably, despite its low bulk density of 68 mg cm^–3^ and high porosity (95%), OPBI-A1 can support a 1 kg load—approximately 2500 times its own weight—without breaking or undergoing significant deformation (Figure 5a). The mechanical properties of OPBI-As were further assessed through uniaxial compression tests (Figure 5b). Interestingly, the mechanical performance of the samples correlate not only with bulk density but also with cross-linking density,^39^ meaning that their mechanical behavior can be tuned through synthesis parameters, such as OPBI precursor concentration and DBX molar ratio, depending on the intended application. The compressive moduli, corresponding to a brief elastic deformation (0.05%–0.25%), remained within a similar range for all OPBI-As, with a slight increase in line with bulk density (0.89–1.27 MPa). However, the strength required for 10% deformation varied significantly among samples, increasing from 0.28 MPa for OPBI-A1 to 0.41 MPa for OPBI-A2 and 0.76 MPa for OPBI-A3 (Table 2). This trend can be attributed to a combination of bulk density and cross-linking content. OPBI-A1 exhibited the lowest compressive strength due to its low density (68 mg cm^–3^), while the differences between OPBI-A2 and OPBI-A3 reflect variations in cross-linking. Although these two samples have nearly identical bulk densities (92 and 93 mg cm^–3^), the higher cross-linking density in OPBI-A3 leads to a more constrained and rigid polymer network. In contrast, the lower cross-linking in OPBI-A2, allows for greater chain mobility and segmental motion, resulting in a softer mechanical response under compression. Nevertheless, all the aerogels exhibited excellent mechanical performance, showing no cracks, crumbling, or pulverization even after 50% deformation (Figure 5c).

**Figure 5:**
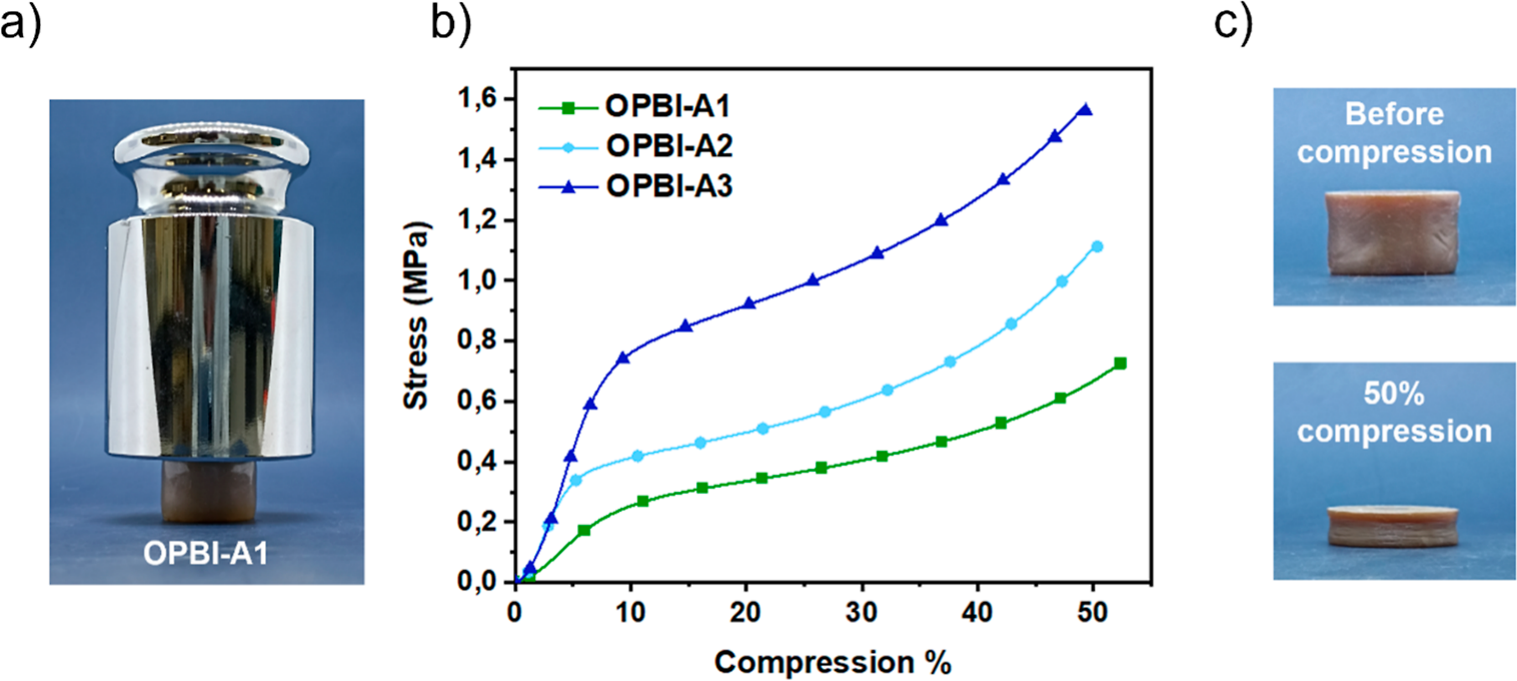
(a) Photograph of OPBI-A1 (ρ_b_ = 68 mg cm^–3^, 95% porosity) supporting more than 2500 times its own weight (1 kg). (b) Stress–deformation curves of OPBI-As. Three identical samples were used for each measurement with their corresponding graphs being presented in Figure S4a. (c) Comparative picture of OPBI aerogel before and after compression to 50% deformation.

## 4 Conclusions

In this work, we successfully developed high-performance polybenzimidazole-based aerogels through the cross-linking reaction of commercially available OPBI copolymer with DBX, yielding thermally insulating materials. These organic aerogels feature low densities of 68–93 mg cm^–3^, high porosity (up to 95%) and substantial specific surface areas (600–700 m^2^ g^–1^). Their thermal conductivity reaches as low as 23.9 mW m^–1^ K^–1^, making them highly effective thermal insulators. In addition, the best performing insulating sample, OPBI-A1, demonstrated exceptional thermal stability, with high decomposition temperatures (Td_1%_ > 500 °C, Td_5%_ > 580 °C) and high char residue (82.6 wt %) at 790 °C, and exhibited flame resistant behavior. The aerogels also exhibit tunable mechanical properties, withstanding significant mechanical stress without breaking or pulverizing, owing to the combination of covalent and noncovalent cross-linking. This unique combination of properties makes OPBI aerogels ideal for extreme thermal environments, with potential applications beyond building insulation, such as protective insulation for EV batteries, lightweight thermal protection suits and space exploration rovers. Their thermal insulating performance, coupled with lightweight and robust mechanical properties, positions them as a leading choice for next-generation high-performance insulation materials.

## Supplementary Material





## Notes

Additional characterization for model compound synthesis, including ^1^H NMR and ^13^C NMR; experimental details for OPBI preparation and supercritical drying; FT-IR spectra; SEM images; stress–deformation curves extended data; Comparative diagram of compressive moduli (PDF)
(MP4)
