# Supplementary material for: Polybenzimidazole Aerogels with High Thermal Stability and Mechanical Performance for Advanced Thermal Insulation Applications
Source: ACS Appl Mater Interfaces. 2025 May 26;17(22):32928–35. doi: 10.1021/acsami.5c05737 (PMC12147065; doi:10.1021/acsami.5c05737)
Supplement: Supplementary file 1 [file am5c05737_si_001.pdf]

Supporting Information

Polybenzimidazole Aerogels with High Thermal Stability and Mechanical Performance for Advanced Thermal Insulation Applications

Christos Pantazidis [a] and Željko Tomović [a]*

[a] Polymer Performance Materials Group, Department of Chemical Engineering and Chemistry, Interactive Polymer Materials (IPM) and Institute for Complex Molecular Systems (ICMS), Eindhoven University of Technology, Eindhoven, MB 5600, The Netherlands.

* Email: z.tomovic@tue.nl

Number of Pages: 8
Number of Figures: 4
Number of Schemes: 1
Number of Tables: 1

Table of contents

Model compound p-bis(benzoimidazolyl)-methylbenzene (BIM) 3
OPBI aerogel preparation 4
Supercritical CO_2 drying setup 4
Extended SEM data 6
Extended data for mechanical properties 7
References 8

Model compound p-bis(benzoimidazolyl)-methylbenzene (BIM)

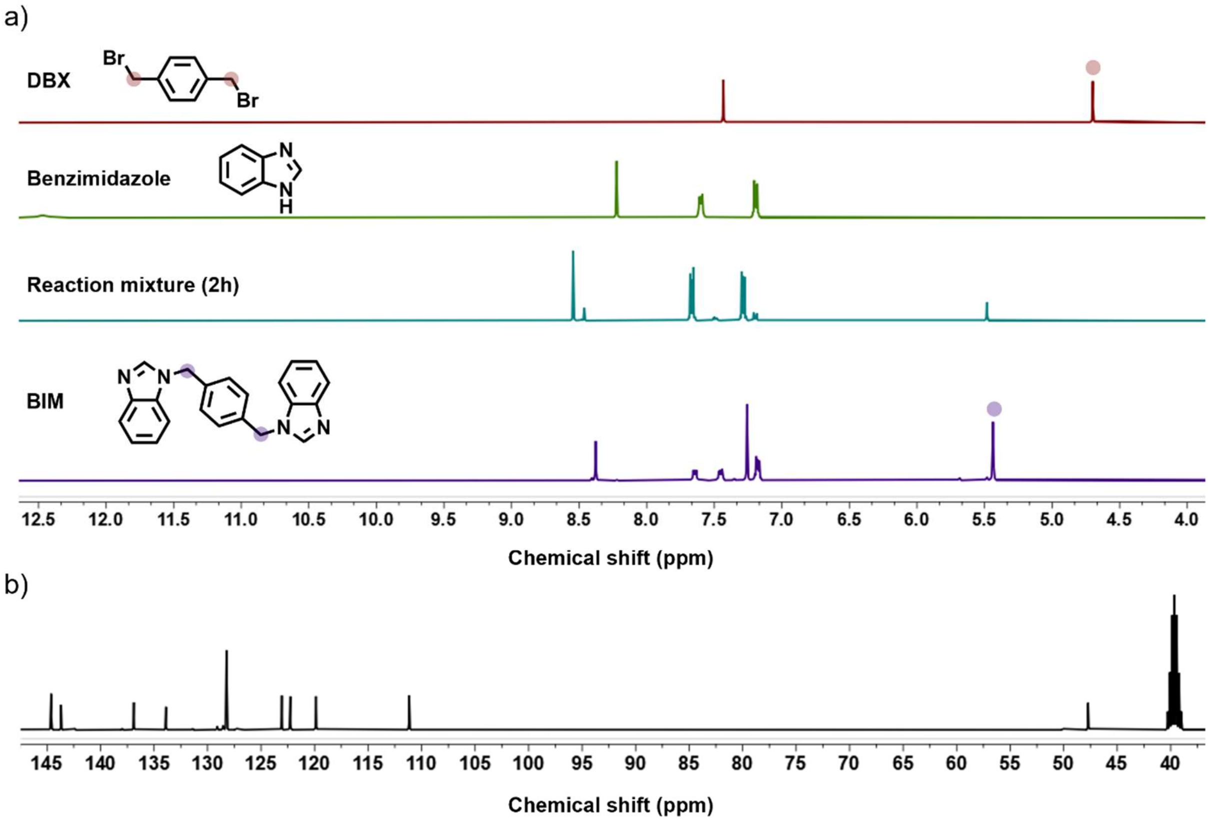

Figure S1. a) ^{1}H NMR spectra of DBX, benzimidazole, reaction mixture after 2h, BIM model compound. b) ^{13}C NMR of BIM model compound.

OPBI aerogel preparation

Table S1. Formulation of OPBI aerogels

Sample Name	OPBI (mg)	DBX (mg)	Solvent	Solvent volume (mL)	OPBI concentration (g mL^{-1})	DBX molar ratio [(a)] (%)	Organogel - Remarks	Shrinkage after drying (%)	Bulk density (mg cm^{-3})	Specific surface area (m^2 g^{-1})
2% w/v OPBI concentration, 5% DBX ratio, **solvent screening**										
OPBI-S1a	200	6.6	**DMSO**	10	0.02	5	Unstable gel	-	-	-
OPBI-S1b	200	6.6	**NMP**	10	0.02	5	Unstable gel	-	-	-
OPBI-S1c	200	6.6	**DMAc**	10	0.02	5	Unstable gel	-	-	-
OPBI-S1d	200	6.6	**DMF**	10	0.02	5	Unstable gel	-	-	-
2% w/v OPBI concentration, 10% DBX ratio, **solvent screening**										
OPBI-S2a	200	13.2	**DMSO**	10	0.02	10	Unstable gel	-	-	-
OPBI-S2b	200	13.2	**NMP**	10	0.02	10	Unstable gel	-	-	-
OPBI-S2c	200	13.2	**DMAc**	10	0.02	10	Unstable gel	-	-	-
OPBI-S2d	200	13.2	**DMF**	10	0.02	10	Unstable gel	-	-	-
4% w/v OPBI concentration, 5% DBX ratio, **solvent screening**										
OPBI-S3a	400	13.2	**DMSO**	10	0.04	5	Unstable gel	-	-	-
OPBI-S3b	400	13.2	**NMP**	10	0.04	5	Soft gel	29	115	318
OPBI-S3c	400	13.2	**DMAc**	10	0.04	5	Soft gel	24	93	466
OPBI-S3d	400	13.2	**DMF**	10	0.04	5	Unstable gel	-	-	-
Samples in NMP, **OPBI concentration (4 and 6 % w/v)** and **DBX ratio (5 and 10 %)** screening										
OPBI-S4a	400	26.4	NMP	10	**0.04**	**10**	Stable gel	18	75	672
OPBI-S4b	600	19.8	NMP	10	**0.06**	**5**	Stable gel	12	87	426
OPBI-S4c	600	39.5	NMP	10	**0.06**	**5**	Stable gel	14	91	597
Samples in DMAc, **OPBI concentration (4 and 6 % w/v)** and **DBX ratio (5 and 10 %)** screening [(b)]										
OPBI-A1	400	26.4	DMAc	10	**0.04**	**10**	Stable gel	16	68	593
OPBI-A2	600	19.8	DMAc	10	**0.06**	**5**	Stable gel	13	92	724
OPBI-A3	600	39.5	DMAc	10	**0.06**	**5**	Stable gel	13	93	624

[(a)] DBX molar ratio is referenced in relation to OPBI monomeric units.
[(b)] The samples discussed in the manuscript.

Supercritical CO_2 drying setup

In the supercritical drying process (SCD), Liquid CO_2 grade 2.7 (purity >99.7%) was used as the exchange agent. The drying setup employs high-pressure extraction/drying units, specifically the "HP-DE200" autoclave system (Scheme S1), provided by Eurotechnica (Germany). This autoclave has a maximum operating temperature of 100 °C and can withstand pressures up to 220 bar. It is equipped with a thermowell containing a NiCr-Ni thermocouple to monitor the internal temperature. Additionally, two venting tubes are installed for solvent extraction and

depressurization. The supercritical drying system includes two thermal baths (Selecta, UNITRONIC 200) for heat exchangers 1 and 2 (4 and 7), a mechanical pump (5) from Maximator, a check valve (2), five needle valves (3, 6, 7, 8, 10, and 11), and a CO2 cylinder (1) (Scheme S1).

The process begins by placing the gels into the autoclave (9) and covering them with the solvent used for gel formation, preventing premature solvent evaporation and minimizing shrinkage. The CO_2 pressure is gradually increased to 100 bar to extract the solvent from the wet gel pores. Heat exchanger 2 (7) maintains the autoclave at a constant temperature of 60 °C. Once supercritical conditions are reached, the solvent is extracted from the gels in the autoclave (9). The CO_2, now enriched with the extracted solvent, is vented out as the autoclave is released, with the pressure maintained by a continuous influx of fresh CO_2. The venting process lasts for approximately 10 to 15 minutes, after which the autoclave is resealed for further extraction. This extraction cycle is repeated three times, with a 30-minute waiting interval between each cycle. Finally, once the aerogel pores are free of solvent, the pressure is gradually released to atmospheric levels through the metering valve over the course of 45 minutes.

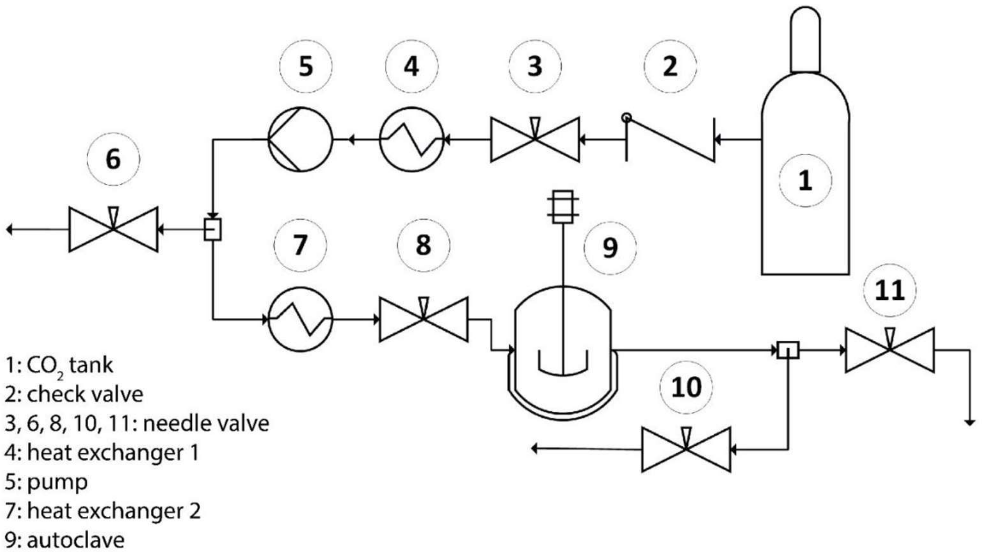

Scheme S1. Flow diagram of supercritical drying setup.

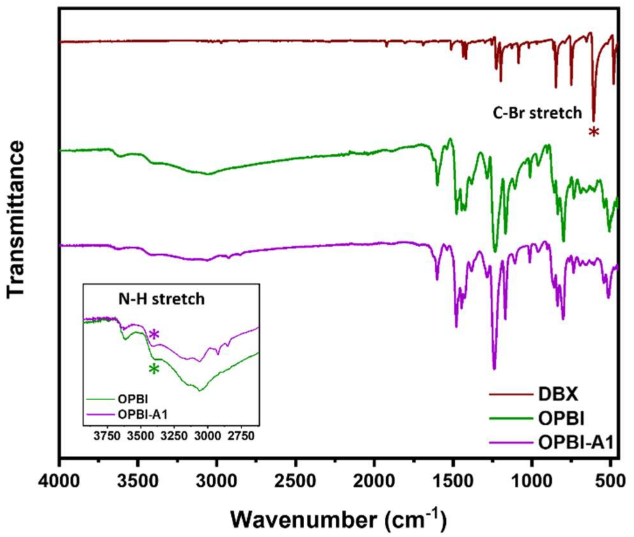

Figure S2. FT-IR spectra of DBX, OPBI copolymer and OPBI aerogel (OPBI-A1 sample).

Extended SEM data

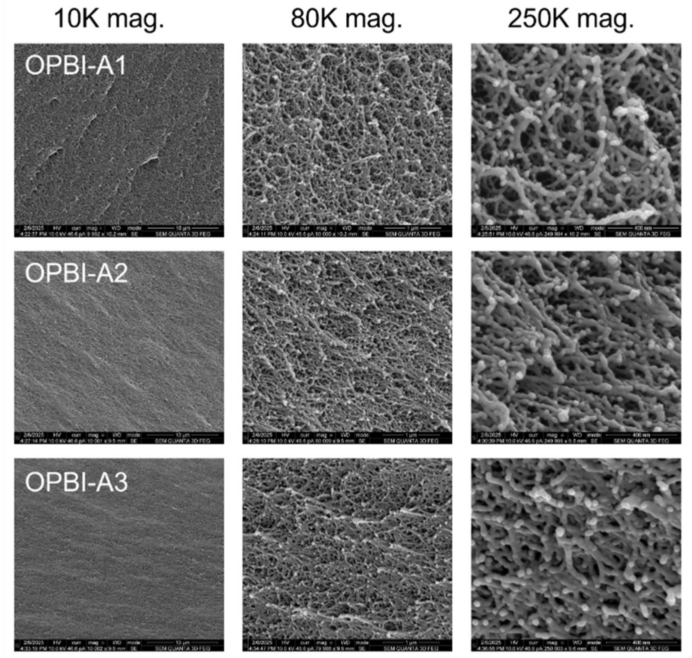

Figure S3. SEM pictures of OPBI-As. Different magnifications are presented (10K, 80K, 250K) to highlight the homogeneity of aerogel morphologies.

Extended data for mechanical properties

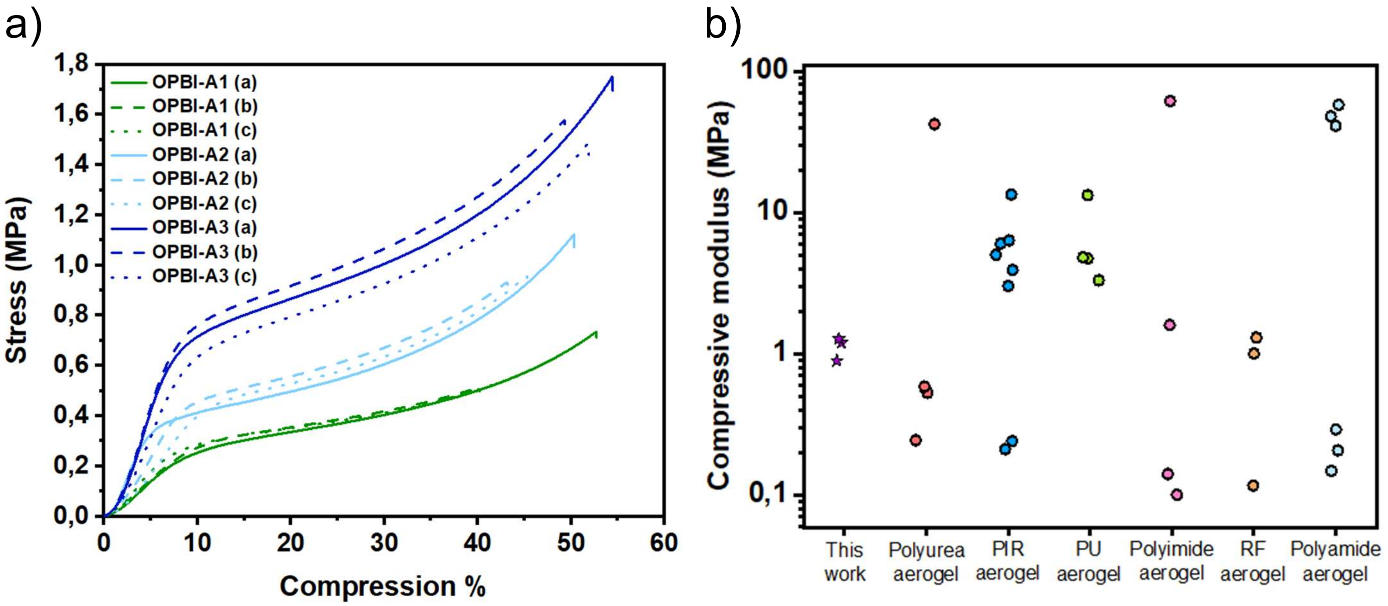

Figure S4. a) Stress - deformation curves of OPBI-As. For each sample, three identical aerogels were prepared and measured. The average values of compressive moduli and compressive strength at 10% deformation are shown in Table 2 of the manuscript. b) Comparison of this works' compressive moduli values with polyurea [1,2], polyisocyanurate (PIR) [3-5] polyurethane (PU) [6,7], polyimide [8-11], resorcinol-formaldehyde (RF) [12,13] and polyamide [14,15] aerogels from literature.

References

[1] J. K. Lee, G. L. Gould, W. Rhine, *J. Sol-Gel Sci. Technol*. **2009**, 49, 209.

[2] A. M. Saeed, C. A. Wisner, S. Donthula, H. Majedi Far, C. Sotiriou-Leventis, N. Leventis, *Chem. Mater.* **2016**, 28, 4867.

[3] T. Taghvaee, S. Donthula, P. M. Rewatkar, H. Majedi Far, C. Sotiriou-Leventis, N. Leventis, *ACS Nano* **2019**, 13, 3677.

[4] N. Leventis, C. Chidambareswarapattar, A. Bang, C. Sotiriou-Leventis, *ACS Appl. Mater. Interfaces* **2014**, 6, 6872.

[5] R. Trifu, G. Gould, S. White, *MRS Adv*. **2017**, 325, 1.

[6] N. Diascorn, S. Calas, H. Sallée, P. Archard, A. Rigacci, *J. Supercrit. Fluids* **2015**, 106, 76.

[7] B. Merillas, F. Villafañe, M. Á. Rodríguez-Pérez, *Nanomaterials* **2022**, 12, 2409.

[8] J. Tian, Y. Yang, T. Xue, G. Chao, W. Fan, T. Liu, *J. Mater. Sci. Technol*. **2022**, 105, 194.

[9] Z. Ma, T. Xue, Q. Wali, Y. Miao, W. Fan, T. Liu, *Compos. Commun*. **2023**, 39, 101528.

[10] O. Tafreshi, S. Ghaffari-Mosanenzadeh, S. Karamikamkar, Z. Saadatnia, S. Kiddell, C. Park, H. Naguib, *J. Mater. Chem. C* **2022**, 10, 5088.

[11] K. Yao, S. Jiang, S. Li, C. Zhang, H. Hou, *Compos. Commun.* **2023**, 38, 101503.

[12] M. Alshrah, M. Tran, P. Gong, H. Naguid, C. Park, *J. Colloid Interface Sci.* **2017**, 485, 65.

[13] M. Schwan, R. Tannert, L. Ratke, *J. Supercrit. Fluids* **2016**, 107, 201.

[14] H. Ren, J. Zhu, Y. Bi, Y. Xu, L. Zhang, *J. Porous Mater.* **2017**, 24, 1165.

[15] J. Williams, M. Meabor, L. McCorkle, C. Mueller, N. Wilmoth, *Chem. Mater.* **2014**, 26, 4163
